# Geriatric Interprofessional Education: In-Person Simulation

**DOI:** 10.1093/geroni/igab046.002

**Published:** 2021-12-17

**Authors:** Diane Brown

**Affiliations:** The University of Akron, Akron, Ohio, United States

## Abstract

Our in-person geriatric interprofessional training model is layered with scaffolds of active learning, tabletop team meeting simulation, assessment of older adult community members at risk for falls, and reflective feedback. The first step addresses knowledge acquisition via online didactic content. The second step reinforces the knowledge gained in the online didactics through in-person posters and interactive skills practice, followed by a profession-specific huddle to communicate patient assessment findings. The third step is an interprofessional team meeting simulation based on a case study of a complex geriatric patient. The fourth step is performing a supervised assessment on an older adult. The assessment incorporates the assessment tools practiced during the poster/skills session and team skills learned in the didactics and simulation. This is followed by the design of an interprofessional team-developed patient-centered plan of care. The event ends with a reflective debrief focused on interprofessional collaborative competencies.

